# Retraction for Shepardson et al., “Induction of Antiviral Immune Response through Recognition of the Repeating Subunit Pattern of Viral Capsids Is Toll-Like Receptor 2 Dependent”

**DOI:** 10.1128/mbio.00430-24

**Published:** 2024-07-12

**Authors:** Kelly M. Shepardson, Benjamin Schwarz, Kyle Larson, Rachelle V. Morton, John Avera, Kimberly McCoy, Alayna Caffrey, Ann Harmsen, Trevor Douglas, Agnieszka Rynda-Apple

## RETRACTION

Vol. 8, no. 6, e01356-17, 2017, https://doi.org/10.1128/mbio.01356-17. The following issues were identified with the data presentation and figure legend in Fig. 5G and H:

1. The figure legend was incorrect in the number of replicates and experiments shown. It was stated that both “panels G and H had five technical replicates per cell line. Each was repeated three times.” Some cell lines did not have five technical replicates each time, and not all cell lines were performed three times; some were only performed twice.
2. For Fig. 5G, source data for one of two experiments (from the TLR1^−/−^ cell line) could not be located.

These errors were identified in response to allegation of research misconduct submitted to Montana State University Office of Research Compliance (MSU ORC) by an anonymous party.

MSU ORC conducted a research misconduct investigation and concluded that both the wording in the figure legend and the fact that we cannot find the source data for the second TLR1^−/−^ cell line experiment presented in this paper were evidence of research misconduct. During the investigation MSU was provided with additional repeat experiments performed in this cell line after publication of this work suggesting that the data published in the manuscript is likely correct. The paper is being retracted to comply with the recommendations of the MSU ORC.

